# Variability of the time course of stimulus-specific adaptation in the inferior colliculus

**DOI:** 10.3389/fncir.2012.00107

**Published:** 2012-12-27

**Authors:** David Pérez-González, Manuel S. Malmierca

**Affiliations:** ^1^Auditory Neurophysiology Laboratory, Institute of Neuroscience of Castilla y León, University of SalamancaSalamanca, Spain; ^2^Department of Cell Biology and Pathology, University of SalamancaSalamanca, Spain

**Keywords:** auditory, stimulus-specific adaptation, inhibition, GABA-A receptor, microiontophoresis, single unit activity

## Abstract

Stimulus-specific adaptation (SSA) is the ability of some neurons to respond better to rare than to frequent, repetitive stimuli. In the auditory system, SSA has been found at the level of the midbrain, thalamus, and cortex. While previous studies have used the whole overall neuronal response to characterize SSA, here we present a detailed analysis on the variations within the time course of the evoked responses. The extracellular activity of well isolated single neurons from the inferior colliculus (IC) was recorded during stimulation using an oddball paradigm, which is able to elicit SSA. At the same time, these responses were evaluated before, during, and after the microiontophoretic application of gabazine, a specific antagonist of GABA_A_ receptors, to study the contribution of inhibition to the responses of these neurons. We then analyzed the difference signal (DS), which is the difference in the PSTH in response to rare and frequent stimuli. We found that, even in a sample of neurons showing strong SSA (i.e., showing larger preference for rare stimuli), the DS was variable and one third of the neurons contained portions that responded significantly better to the frequent stimuli than to the rare. This variability is not observed when averaging the responses of multiple cells. Furthermore, the blockade of GABA_A_ receptors increased the number of neurons showing portions that responded better to the frequent stimuli, indicating that inhibition in the IC refines and sharpens SSA in the neural responses.

## Introduction

Since the original paper by Ulanovsky and colleagues (Ulanovsky et al., 2003), a number of studies have appeared in the last decade exploring the phenomenon so-called stimulus-specific adaptation (SSA), which consists on the ability of some neurons to reduce their responses to repetitive stimuli, while keeping their responsiveness to different, albeit similar, stimuli. SSA is elicited by presenting the stimuli in an oddball paradigm (Ulanovsky et al., 2003), in which each trial of a sequence contains one of two different stimuli, which have different probabilities of occurrence. The one appearing with the higher probability is referred to as the standard stimulus, while the other, at the lower probability, is known as the deviant stimulus. The frequent repetitions of the standard cause a gradual reduction of the response on these neurons, but whenever a deviant appears, the neurons are still able to respond promptly and robustly. SSA has been suggested to play a role in auditory scene analysis (Winkler et al., 2009), attention (Escera et al., 1998; Fritz et al., 2007), and novelty or change detection (Jääskeläinen et al., 2004; Nelken and Ulanovsky, 2007; Slabu et al., 2010; Grimm et al., 2011; Gutfreund, 2012).

Thus far, SSA has been found at the levels of the auditory midbrain (Pérez-González et al., 2005, 2012; Malmierca et al., 2009a; Lumani and Zhang, 2010; Zhao et al., 2011; Anderson and Malmierca, 2012; Patel et al., 2012), thalamus (Anderson et al., 2009; Yu et al., 2009; Antunes et al., 2010; Antunes and Malmierca, 2011), and cortex (Ulanovsky et al., 2003, 2004; von der Behrens et al., 2009; Farley et al., 2010; Taaseh et al., 2011; Yaron et al., 2012). The inferior colliculus (IC) is the main auditory center of the midbrain, acting as a site for convergence of most ascending auditory inputs (Oliver and Shneiderman, 1991; Malmierca, 2003) and where auditory information is integrated by combining the multiple excitatory and inhibitory projections arising from lower nuclei, the auditory cortex, as well as commissural and intrinsic connections (Malmierca, 2003; Malmierca et al., 2009b; Malmierca and Ryugo, 2011, 2012). Therefore, the IC stands as a unique auditory center that combines the attributes necessary for the formation of functional microcircuits. In turn, the IC sends the processed auditory information to the cortex, through the thalamus. Inhibition, in particular that mediated by GABA, has been found to play a key role in the sound processing that takes place in the IC (LeBeau et al., 1996; Wu et al., 2004; Gittelman et al., 2012), regulating several properties including sound intensity (Sivaramakrishnan et al., 2004), amplitude modulation (Caspary et al., 2002; Zhang and Kelly, 2003), frequency selectivity (Palombi and Caspary, 1996; Koch and Grothe, 1998; LeBeau et al., 2001), sound localization (Ingham and McAlpine, 2005), or sound duration (Casseday et al., 1994, 2000; Covey et al., 1996). SSA has been found to be a unique property of the non-lemniscal subcortical nuclei (Malmierca et al., 2009a; Antunes et al., 2010; Antunes and Malmierca, 2011; Duque et al., 2012). In this respect, it should be emphasized that there are between 10 and 20 times more GABAergic than glycinergic puncta (Merchán et al., 2005) in the dorsal and lateral cortical areas of the IC, i.e., in the non-lemniscal areas, which suggest that in these IC regions GABA plays a key role in the processing of auditory stimuli through inhibition.

While SSA is commonly estimated from the whole evoked response of the neuron, several studies have described its variations across the response, either based on single unit recordings (Ulanovsky et al., 2003; Malmierca et al., 2009a; von der Behrens et al., 2009; Zhao et al., 2011), multiunit recordings (Ulanovsky et al., 2003; von der Behrens et al., 2009; Farley et al., 2010; Bäuerle et al., 2011), or local field potentials (LFPs) (von der Behrens et al., 2009; Patel et al., 2012). However, these studies tend to report the aggregated variations at the population level, a fact that will not reflect the details in the temporal variability on the responses of single neurons. We have used a data set that was published previously with a different purpose (Pérez-González et al., 2012), in order to perform a detailed analysis of the time course of the responses evoked during SSA. The original data set comprises single neurons located in the dorsal, lateral, and rostral cortices of the IC (Malmierca et al., 1993, 2011). Neurons located in those regions display a high degree of SSA. These neurons were tested for SSA using an oddball paradigm while blocking local GABA_A_ receptors, using the powerful technique of microiontophoresis, which allows examining the contribution of inhibition to the neuronal responses.

## Materials and methods

### Data set and data collection

We analyzed a data set comprising extracellular recordings of 46 single units from the IC of Long Evans adult rats. The neurons were located in the dorsal, lateral, and rostral cortices of the IC, which display a large amount of SSA (Pérez-González et al., 2005; Malmierca et al., 2009a). All cases from the original set were included in the analysis. The original data were collected and analyzed for other purposes (Pérez-González et al., 2012); therefore, here it suffices a brief description of the most relevant aspects of the data collection.

The experiments were conducted on urethane-anesthetized animals, which were placed in a stereotaxic frame and subjected to closed-field auditory stimulation (Rees, 1990; Rees et al., 1997; Malmierca et al., 2008, 2009a,b). A craniotomy was performed to allow for access to the IC through the cortex. The extracellular activity of single neurons in the IC was recorded with tungsten electrodes, and the spike times were stored for further analysis. The recording electrodes were attached to multibarrel pipettes, filled with either 20 mM gabazine (SR-95531, Sigma-Aldrich) or 1 mM NaCl for current compensation. Gabazine is a selective antagonist of GABA_A_ receptors, which lacks the side effect on calcium-dependent potassium channels of bicuculline, the other typical antagonist of GABA_A_ receptors (Kurt et al., 2006). Gabazine was released by microiontophoresis (Neurophore BH-2 System, Harvard Apparatus), applying currents to the pipettes (typically 40–50 nA), to locally block the GABA_A_ receptors at the recording site. Controls were conducted to rule out current artifacts. Data were collected before (*control condition*), during (*gabazine condition*), and after (*recovery condition*, not analyzed here) the application of gabazine, and comparisons were made between the different conditions.

**Figure 1:**
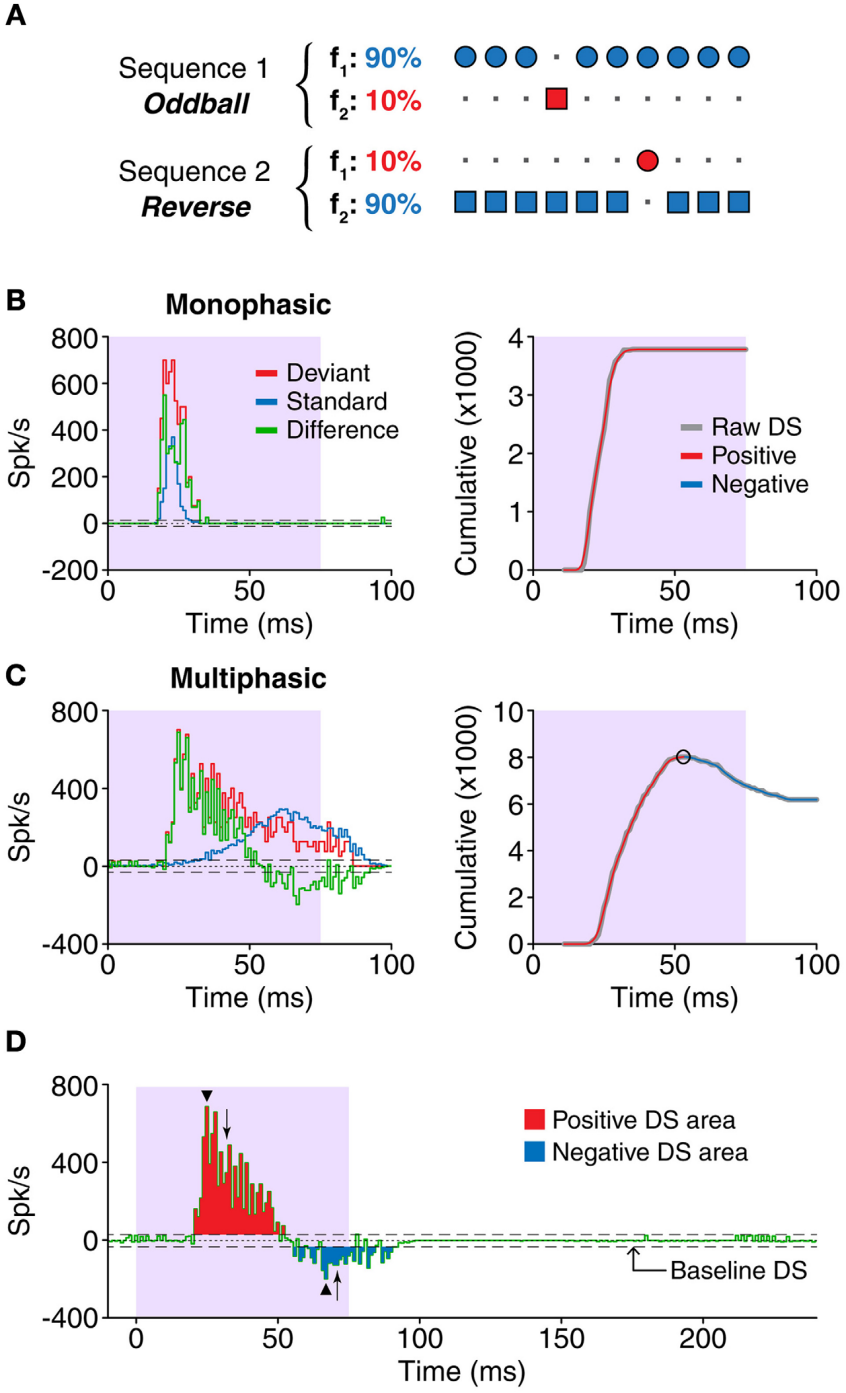
**(A)** In the oddball paradigm, a sequence is built containing two different stimuli, in this case, two pure tones at different frequencies (f_1_, f_2_). The stimuli are equally spaced, but their probabilities are different. One of the tones, the standard (blue), appears in 90% of the trials, while the other, called deviant (red), appears in the remaining 10%. A complimentary sequence is presented afterwards, with the probabilities of the stimuli reversed. Thus, comparisons can be drawn for each of the tones when they are presented as deviants or standards. **(B,C)** Left panels show examples of responses (PSTH) to one tone presented as standard (blue) or deviant (red). The difference signal (DS, green) was calculated as deviant–standard. We classified the shapes of the DS as ***monophasic* (B)** or ***multiphasic* (C)**. The bin size for all the histograms in this study was 1 ms. The shaded background indicates the duration of the stimulus. Right panels show the cumulative functions of each corresponding DS. The raw cumulative sum function (gray) was smoothed, and the peaks and valleys (circles) were found (see “Materials and Methods”). The increasing sections between peaks (red) indicate portions were the DS is positive, while the decreasing sections indicate the negative portions of the DS. The extent of the negative portions was used to determine the type of DS. **(D)** Only DS values larger than the baseline DS were considered for the study. The positive (red) and negative (blue) portions of the DS were measured separately. The measurements included the latency and magnitude of the peaks (arrowheads), the duration and total area of each portion (red and blue areas), and the latency of the median spike (arrows). The horizontal dashed lines represent the threshold for the baseline activity, i.e., random fluctuations of the DS (mean + 3SD).

Acoustic stimuli were delivered monaurally through calibrated speakers (model EC1, TDT; Tucker-Davis Technologies), to the ear contralateral to the recording side. Stimulation and recording was performed by TDT System II equipment, controlled with custom software. Search stimuli included white noise and pure tones whose frequency was changed often to reduce neuronal adaptation. The experimental stimuli were pure tones with duration of 75 ms, including 5 ms rise/fall ramps, at a rate of 4/s. The frequency response area (FRA) of the neuron was determined using an automated procedure (Duque et al., 2012; Pérez-González et al., 2012) and was used to establish the best frequency (the frequency that evoked a response at the lowest intensity) and the threshold (the lowest intensity able to evoke a response). The stimuli were chosen to fall within the response area of the neuron. For each neuron, a pair of pure tones of different frequencies (f_1_ and f_2_; 0.36–0.53 octaves apart, relative to the lowest frequency of the pair) that elicited a similar firing rate at the same sound level (10–40 dB above threshold) were presented in an oddball paradigm (Näätänen, 1992; Ulanovsky et al., 2003, 2004; Malmierca et al., 2009a; Duque et al., 2012). This paradigm consisted of a sequence of 400 stimuli containing either frequency in a probabilistic manner. One frequency (f_1_) was presented as the standard (i.e., high probability within the sequence, 90%); interspersed randomly among the standards were the deviant stimuli (i.e., low probability, 10%) at the second frequency (f_2_). Afterwards the relative probabilities of the two stimuli were reversed, with f_2_ as the standard and f_1_ as the deviant (e.g., Figure 1A). The original and the reverse sequence were alternated during the whole course of the experiment.

### Data analysis

The spike times in response to the oddball paradigm were used to calculate peristimulus-time histograms (PSTH) separately for the deviant and the standard stimuli (e.g., Figures 1B,C, red and blue traces, respectively). The width of the PSTH bins was 1 ms. For each neuron we obtained eight PSTH: two frequencies (f_1_, f_2_), at two probabilities (10% for deviants, and 90% for standards), in two conditions (*control* and *gabazine*, i.e., before and during the application of gabazine). Due to the different probabilities, the PSTH were derived from different numbers of trials (40 trials in the case of the deviants and 360 trials in the case of the standards). The firing rates were expressed as spikes per second (spk/s) in order to properly compare the responses.

The PSTH of the neurons was classified based on the firing pattern. After correcting for spontaneous activity, the PSTH of a neuron was considered onset if 99% of the response was included in a 30 ms window; otherwise it was considered sustained. For simplicity, we did not perform a more detailed classification of the firing pattern. The 30-ms limit for onset responses has been used previously for neurons in the IC (LeBeau et al., 1996; Lumani and Zhang, 2010), while the 99% criterion avoids misclassification of neurons with spurious late spikes that otherwise would be considered purely onset. Since the firing pattern has been found to depend on the type of stimulus (Pérez-González et al., 2012), this classification was done separately for the responses to deviants and to standards. We used a two-letter nomenclature to define the response of each neuron, the first letter corresponding to the firing pattern in response to standards, and the second in response to deviants. This way, neurons were classified as O-S when the standard was onset but the deviant was sustained, S-O when only the deviant was onset, O-O when both the deviant and the standard were onset and S-S when both were sustained.

The difference signal (DS) was calculated as the difference (in a per-bin basis) between the PSTH for the deviants and the standards (PSTH_deviant_ – PSTH_standard_) in response to the same frequency, in the same condition (e.g., Figures 1B,C, green trace). Thus, positive values of the DS indicate that the response to a stimulus when deviant was larger than the response to the same stimulus when standard, and vice versa.

All the calculations based on the DS were corrected for the baseline fluctuations related to the spontaneous activity (i.e., the activity outside of the evoked response), calculated from a window of 50 ms at the end of each trial. The DS from this time window was obtained, the absolute value of those bins was calculated, and the mean + 3SD was used as threshold for separating the baseline activity. Only the bins whose absolute value was larger than this figure were considered to be significantly different to the baseline DS. In the calculations regarding the magnitude of the DS, this value was subtracted from each significant bin.

We categorized each DS based on its fluctuations during the course of the response. For this purpose, we calculated the cumulative function of the DS (after correcting for baseline activity) and applied a moving average filter (2 passes, 3-bin wide) to smooth high frequency variations. In this cumulative function, the increasing sections reflect positive DS values while the decreasing ones represent the negative DS values. To differentiate the positive from the negative sections, we identified the peaks and valleys of the function, excluding those smaller than 5% of the average firing rate of the neuron. This way, we classified the DSs as ***monophasic***, when the corresponding cumulative function did not contain such peaks or valleys, or ***multiphasic***, in the opposite case. Examples of neurons with monophasic and multiphasic DSs are shown in Figures 1B,C, respectively, as well as the cumulative functions that illustrate the classification process.

We performed a number of calculations in order to characterize the timing and magnitude of the positive and negative portions of the DS, on each neuron. After removing the baseline activity, we applied a time window that included only the evoked response, as judged by visual inspection of the PSTH. Since the response latency and duration were very variable, we considered this time window on a case-by-case basis, as we think this approach is more appropriate than establishing a fixed window for all the cases. First, we calculated the number of positive and negative bins as estimation of the duration of each portion (Figure 1D, red and blue bins, respectively). The area of the positive or negative bins (Figure 1D, red and blue areas, respectively), indicated the magnitude of each portion, and their timing was estimated from the latency of the median spike, relative to the onset of the stimulus (Figure 1D, arrows). On the other hand, we also calculated parameters relative to the peak of each portion (i.e., the bin with the largest positive or negative magnitude; Figure 1D, arrowheads), in particular the latency and the firing rate. For neurons with multiple positive or negative peaks, these calculations were based on the largest positive or negative peak, respectively. All the calculations were corrected for the baseline activity (Figure 1D, dashed line) as described above. Differences between groups were compared using either a Two-Way ANOVA test [Figures 3C, 4C, 5C, 6C; factors: sign of DS (positive or negative) and condition (control or gabazine) and Figures 3G,H, 4G,H, 5G,H, 6G,H; factors: sign of DS (positive or negative) and firing pattern (S-S, S-O, O-S, or O-O)] or a Three-Way ANOVA test [Figures 3D, 4D, 5D, 6D; factors: sign of DS (positive or negative), type of DS (monophasic or multiphasic) and condition (control or gabazine)] and corrected for multiple comparisons using the Tukey–Kramer method. Results were considered significant when *p* < 0.05.

The probability of occurrence of a positive or negative DS was calculated as the percentage of cases that contained a significantly positive or negative DS value in each particular bin. The results of this calculation are illustrated on Figure 7.

## Results

We calculated the PSTH of the responses of 46 neurons in the IC tested in an oddball paradigm, before and during the iontophoretic application of the GABA_A_ antagonist gabazine. Each of the frequencies presented in the oddball paradigm for each neuron was considered separately for the analyses, so the number of cases is 92 frequencies. For each of these cases we calculated the DS, as the difference between the PSTH for deviants and standards (Figures 1B,C). The DS is an indicator of the parts of the response that prefer the deviant stimuli (positive DS) or the standard ones (negative DS).

### PSTH of the population

**Figure 2:**
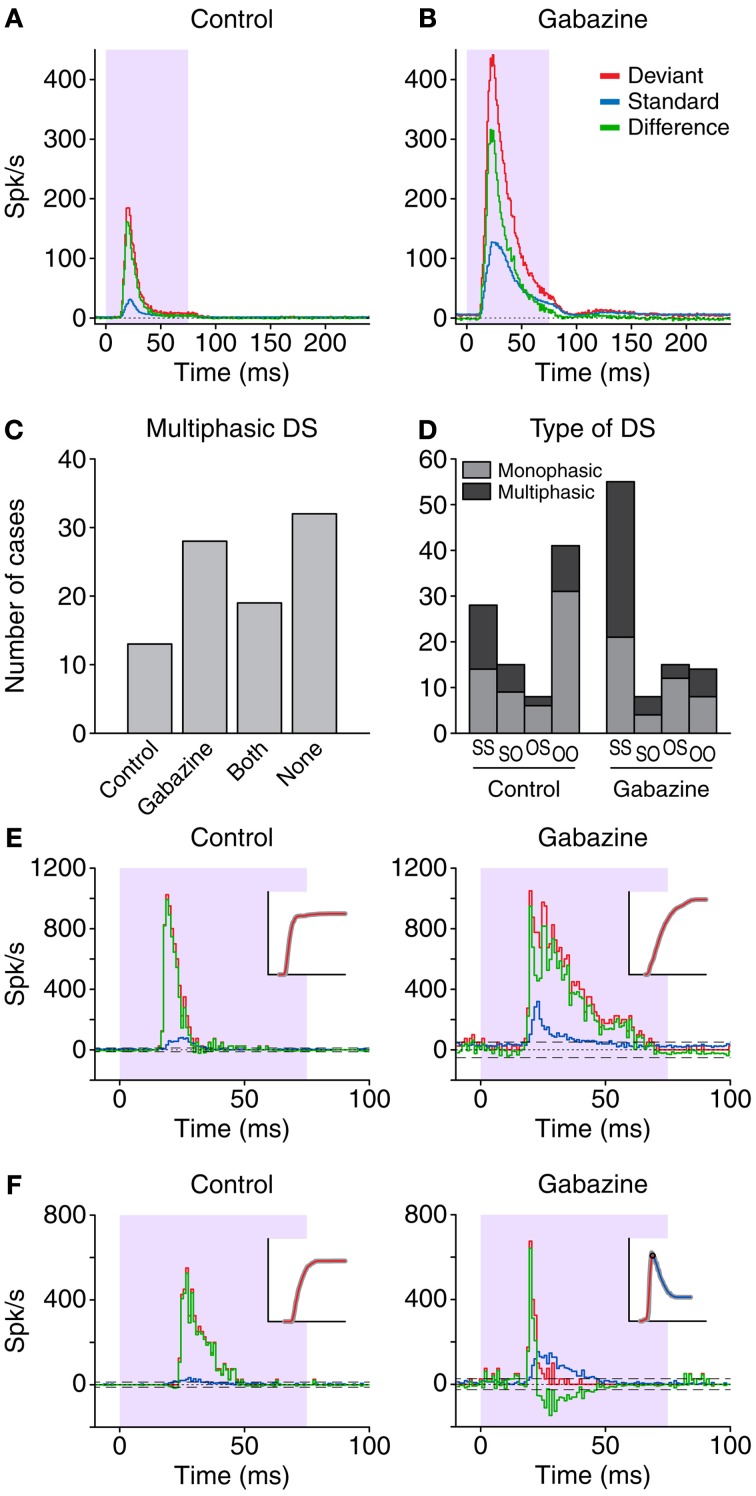
**Grand average PSTH from the sample of neurons in the control (A) and gabazine (B) conditions.** The red lines correspond to the responses to the deviant stimuli, while the blue lines represent the responses to the standard stimuli. The difference signal (DS; deviant-standard) is represented in green. The shaded background indicates the duration of the stimulus. The length of the histogram bins is 1 ms. **(C)** Distribution of the cases were the DS was classified as multiphasic. The *Control* and *Gabazine* groups indicate the number of cases that only presented a negative DS during one of those conditions. *Both* indicates the neurons that had a negative part in both conditions, while neurons that did not have a negative portion under any condition are labeled as *None*. **(D)** Distribution of the cases based on the firing pattern. *S-S*, both standard and deviant were sustained; *S-O*, only the deviant was onset; *O-S*, only the standard was onset; *O-O*, both the deviant and the standard were onset. **(E,F)** Examples from two different neurons during the control and gabazine conditions. In both cases, the DS type is monophasic during the control condition, but in **(F)** the DS type transforms to multiphasic during the gabazine condition. The insets show the cumulative function of the DS, used for the classification.

As a first approximation, we calculated the grand average of all the responses, for all the neurons (Figure 2). During the control condition (Figure 2A), this resulted in a mainly onset PSTH, with a low-firing sustained part, for both standards and deviants. The peak response to the deviants (184.8 spk/s) was considerably larger than the peak response to the standards (31.2 spk/s). The latencies of the peak response for the deviant and the standard were similar (29.5 and 31.5 ms, respectively), although it has been shown that in individual neurons the latency in response to deviants is significantly shorter than in response to standards (Malmierca et al., 2009a; Antunes et al., 2010; Duque et al., 2012; Pérez-González et al., 2012). The resulting DS was positive during the whole evoked response (peak: 161.3 spk/s; peak latency: 28.5 ms). In the gabazine condition (Figure 2B) the peak rates were much larger in response to both deviant (441.3 spk/s) and standard stimuli (127.4 spk/s), but the response to the deviant stimuli remained substantially larger than the one to the standard stimuli. The shape of the PSTH became broader in both cases, while the latencies for the peak response were slightly longer (33.5 ms for the deviant; 32.5 ms for the standard). The DS of the population grand average was also positive for the duration of the evoked response (peak: 316.2 spk/s; peak latency: 31.5 ms). Together, the data from the averaged responses are consistent with the previously reported effect of GABA_A_-mediated inhibition (Pérez-González et al., 2012), wherein inhibition would modify the contrast between responses to standard and deviant by providing a control of the response gain, in what is known as “iceberg effect” (Rose and Blakemore, 1974; Isaacson and Scanziani, 2011).

While the DS was always positive in the averaged population response, this was not the case in the individual responses. All the individual DS had a positive part, but 34.8% (32/92) in the control condition also had significant negative portions, and were thus classified as multiphasic. In the gabazine condition, 51.1% (47/92) of the cases were classified as multiphasic. Not all the neurons that were multiphasic in the control condition maintained this classification during the gabazine condition; only 20.7% (19/92) of the cases were multiphasic during both control and gabazine conditions, while 14.1% (13/92) were multiphasic only during the control condition and 30.4% (28/92) only during the gabazine condition (Figure 2C). Thirty-five percent (32/92) of the cases did not show a negative part during any of the conditions.

Figure 2D shows the distribution of the types of DS and the firing patterns. During the control condition, the most common combination was monophasic neurons with onset patterns for both types of stimuli, deviant and standard (group O-O, 31/92). In contrast, during the gabazine condition, the most common combination was multiphasic neurons with sustained patterns in response to both deviants and standards (group S-S, 34/92). Figures 2E,F show the responses of two different neurons. Both of them displayed a DS (green trace) categorized as monophasic in the control condition (left panels), but the application of gabazine (right panels) modified substantially the response of the neuron in Figure 2F, to the point that the DS became multiphasic (see insets).

### Duration of the difference signal

**Figure 3:**
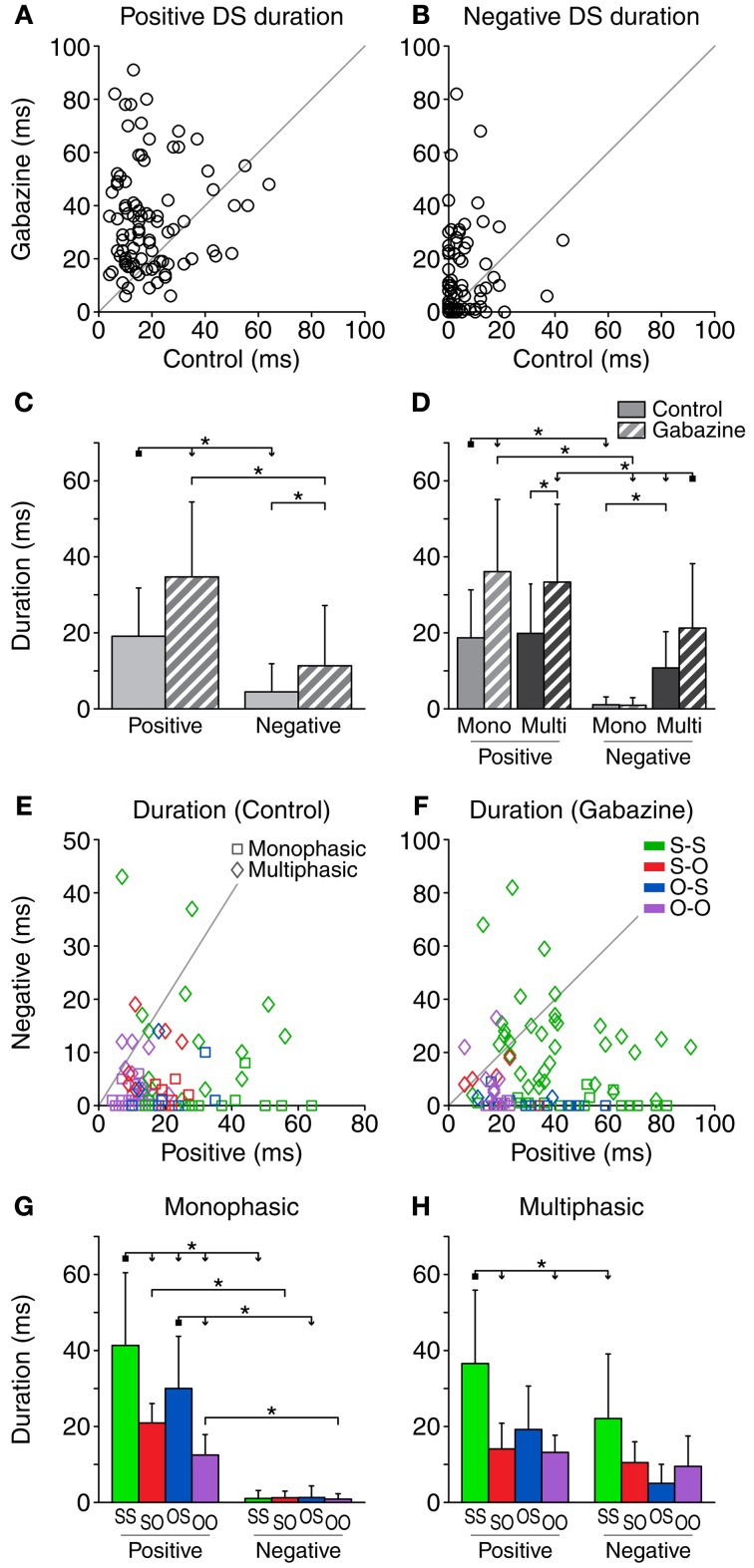
**Duration of the DS (number of 1-ms bins where the response is significantly larger than the baseline DS).** The duration of both the positive **(A)** and negative **(B)** parts of the DS increased during the application of gabazine. **(C)** Mean and standard deviation of the duration for all the cases pooled together, in the control (solid fill) and gabazine (pattern fill) conditions. **(D)** Mean and standard deviation of the duration, grouped by DS type. The duration of the negative part of the DS was shorter than the duration of the positive part for most neurons, both in the control **(E)** and the gabazine **(F)** conditions. The axes in these panels have been plotted at different scales for a better visualization of the data. **(G)** Mean and standard deviation of the different firing pattern groups for the monophasic or **(H)** multiphasic units, including units in both the control and gabazine conditions. *Mono*, monophasic; *Multi*, multiphasic. *S-S*, standard and deviant sustained; *S-O*, standard sustained and deviant onset; *O-S*, standard onset and deviant sustained; *O-O*, standard and deviant onset. Asterisks indicate *p* < 0.05. For all the scatter plots in this and similar figures, *n* = 92 unless stated otherwise. In this and similar figures, some of the significance brackets have been collapsed into a complex bracket, to reduce clutter. They indicate significant differences (*p* < 0.05) between the group at the square end and every group under an arrowhead.

The duration of the positive and negative parts of the DS was estimated as the number of 1-ms bins which were significantly different from the baseline DS. The duration of the positive parts in the control condition was on average 19.1 ± 12.7 ms, and increased to 34.7 ± 19.7 ms in the gabazine condition (Figure 3A). The application of gabazine caused an increment of the duration of the positive part on 75% of the cases (69/92), while 24% of the cases (22/92) showed a decrement. Taking all the cases into account, the average increment of the duration of the positive parts during the gabazine condition was 15.6 ± 22.8 ms. On the other hand, the duration of the negative parts was only 4.4 ± 7.4 ms in the control condition and 11.3 ± 15.9 in the gabazine condition (Figure 3B). Only 50% (46/92) of the cases showed an increment of the duration of the negative parts during the gabazine condition, while 27% of the cases (25/92) showed a decrement. In 3 cases (3%), the duration of the negative parts was the same in the control and gabazine conditions. In 18 cases (20%), there was no negative part during either the control or the gabazine conditions. Taking all the cases into account, the average increment of the duration of the negative parts during the gabazine condition was 6.9 ± 16.3 ms.

When pooling all cases together (Figure 3C), we found significant differences between the duration of the positive and the negative parts, in both the control and the gabazine conditions. In addition, the duration of both parts was significantly increased during the gabazine condition. We did a similar analysis sorting the cases into DS and firing pattern types (Figure 3D). We found that the duration of the positive parts of both the monophasic and multiphasic types was significantly affected by gabazine, but the duration of the negative parts was only affected by gabazine in the case of the multiphasic units. The duration of the positive parts was very similar for monophasic and multiphasic neurons, but the duration of the negative parts of the monophasic units was significantly smaller than that of the multiphasic units. In addition, the duration of the positive parts was significantly larger than the duration of the negative parts in all the groups but the multiphasic/control.

The duration of the positive part of the DS was longer than the negative part in most of the cases [93.5% (86/92) during the control condition and 84.8% (78/92) during the gabazine condition], as shown in Figures 3E,F. On average, the positive part of the DS was 14.6 ± 14.1 ms longer than the negative part in the control condition. This difference increased notably in the gabazine condition, where the positive part was 23.4 ± 25.8 ms longer than the negative.

A detailed analysis of the firing pattern groups, including units in both the control and gabazine conditions, showed that the S-S group had the longest positive DS duration. For the monophasic units, all the groups had longer positive durations than negative (Figure 3G), while for the multiphasic units only the S-S group had significantly different positive and negative DS durations (Figure 3H).

### Area of the parts of the difference signal

**Figure 4:**
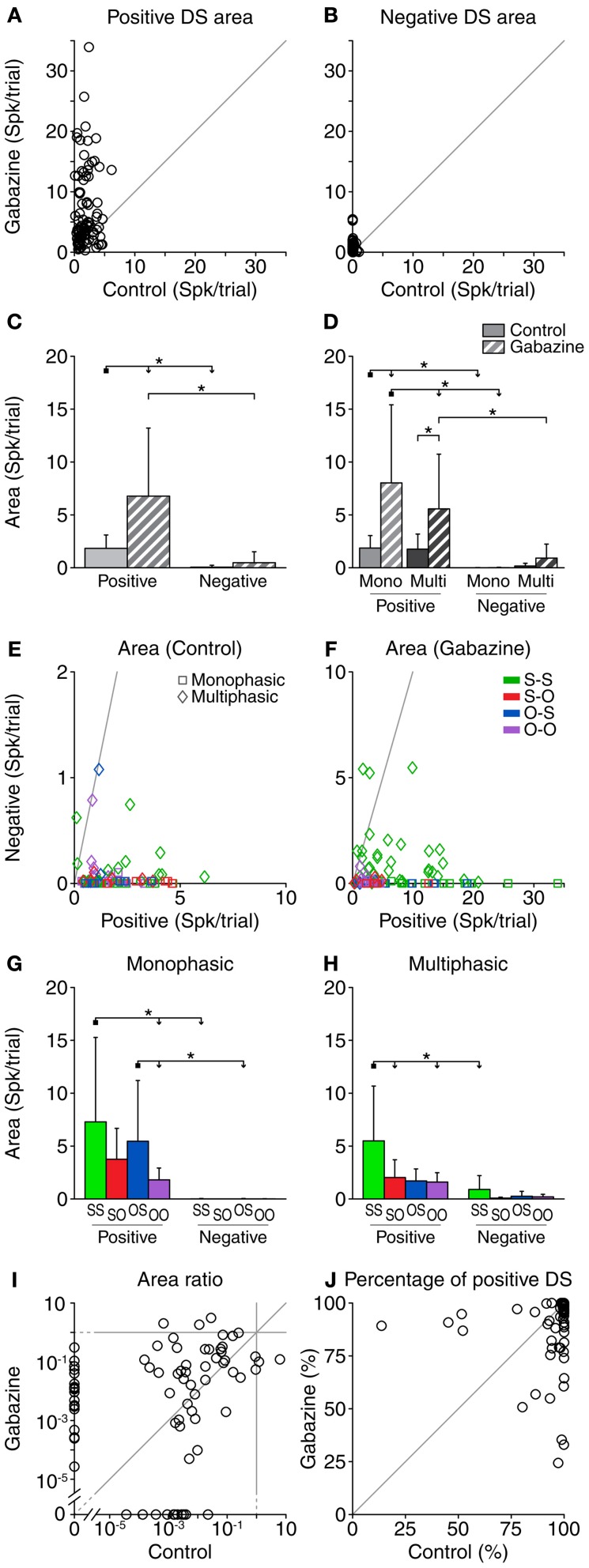
**Area (accumulated response) of the DS for each neuron, corrected for the baseline DS.** The area of both the positive **(A)** and negative **(B)** portions of the DS increased during the gabazine condition. **(C)** Mean and standard deviation of the area for all the cases pooled together, in the control (solid fill) and gabazine (pattern fill) conditions. **(D)** Mean and standard deviation of the area, grouped by DS type. The area of the positive portions of the DS was larger than that of the negative ones for most neurons, both in the control **(E)** and the gabazine **(F)** conditions. The axes in these panels have been plotted at different scales for a better visualization of the data. **(G)** Mean and standard deviation of the different firing pattern groups for the monophasic or **(H)** multiphasic units, including units in both the control and gabazine conditions. The area ratio (negative area/positive area) **(I)** and the percentage of positive area **(J)** for each individual DS show that, while the amount of positive and negative DS portions changed during the gabazine condition, there was no clear trend. *Mono*, monophasic; *Multi*, multiphasic. *S-S*, standard and deviant sustained; *S-O*, standard sustained and deviant onset; *O-S*, standard onset and deviant sustained; *O-O*, standard and deviant onset. Asterisks indicate *p* < 0.05.

We calculated the total area of the positive and negative parts of the DS for each stimulus (e.g., red and blue areas in Figure 1D). The average area of the positive DS parts was 1.8 ± 1.3 spk per trial in the control condition, and in increased to 6.77 ± 6.44 spk per trial in the gabazine condition (Figure 4A). Gabazine caused an increment of the area of the positive DS in 85% (78/92) of the cases, and a decrement in the resting 15% (14/92). Taking all the cases into account, the area of the positive DS parts increased an average of 4.9 ± 6.4 spk per trial in the gabazine condition. The area of the negative DS in the control condition was only 0.06 ± 0.17 spk per trial, while in the gabazine condition it increased up to 0.47 ± 1.0 spk per trial (Figure 4B). Only 54% of the cases (50/92) showed a larger negative area in the gabazine condition relative to the control condition, while in 25% of the cases (23/92) the negative area was smaller. In 1 case (1%), the area of the negative DS parts was the same in the control and gabazine conditions. In 18 cases (20%), there was no negative part during either the control or the gabazine conditions. Taking all the cases into account, the average increment of the area of the negative parts during the gabazine condition was 0.41 ± 1.05 ms.

When all cases were pooled, gabazine only had a significant effect on the area of the positive parts of the DS (Figure 4C). Nevertheless, the positive area was significantly larger than the negative in both the control and gabazine conditions. When sorting the cases in DS and firing pattern types (Figure 4D), the results were similar to the analysis of the duration of the DS parts. The positive DS area increased significantly for the monophasic and multiphasic groups, but not the negative DS area. Likewise, the positive DS area was significantly larger than the negative DS area for the monophasic group (in both control and gabazine conditions), but for the multiphasic group it was larger only during the gabazine condition.

The area of the positive DS was larger than the area of the negative DS in almost every case, both in the control (98%, 90/92) and the gabazine conditions (97%, 89/92). Only 2 cases in the control condition and 3 cases in the gabazine condition showed a negative DS larger than the positive DS (Figures 4E,F).

Not surprisingly, the area of the negative DS parts was negligible for the monophasic units. The largest positive and negative DS areas corresponded to the neurons classified as S-S, while the smallest DS areas were those of the neurons classified as O-O (Figures 4G,H).

The area ratio (negative area/positive area) was larger in the gabazine condition than in the control condition for 64% (47/74) of the cases (Figure 4I). The area of the positive DS accounted for 95.8 ± 12.9% of the total DS area in the control condition, and 91.5 ± 15.6% in the gabazine condition (Figure 4J).

### Peak rate of the difference signal

**Figure 5:**
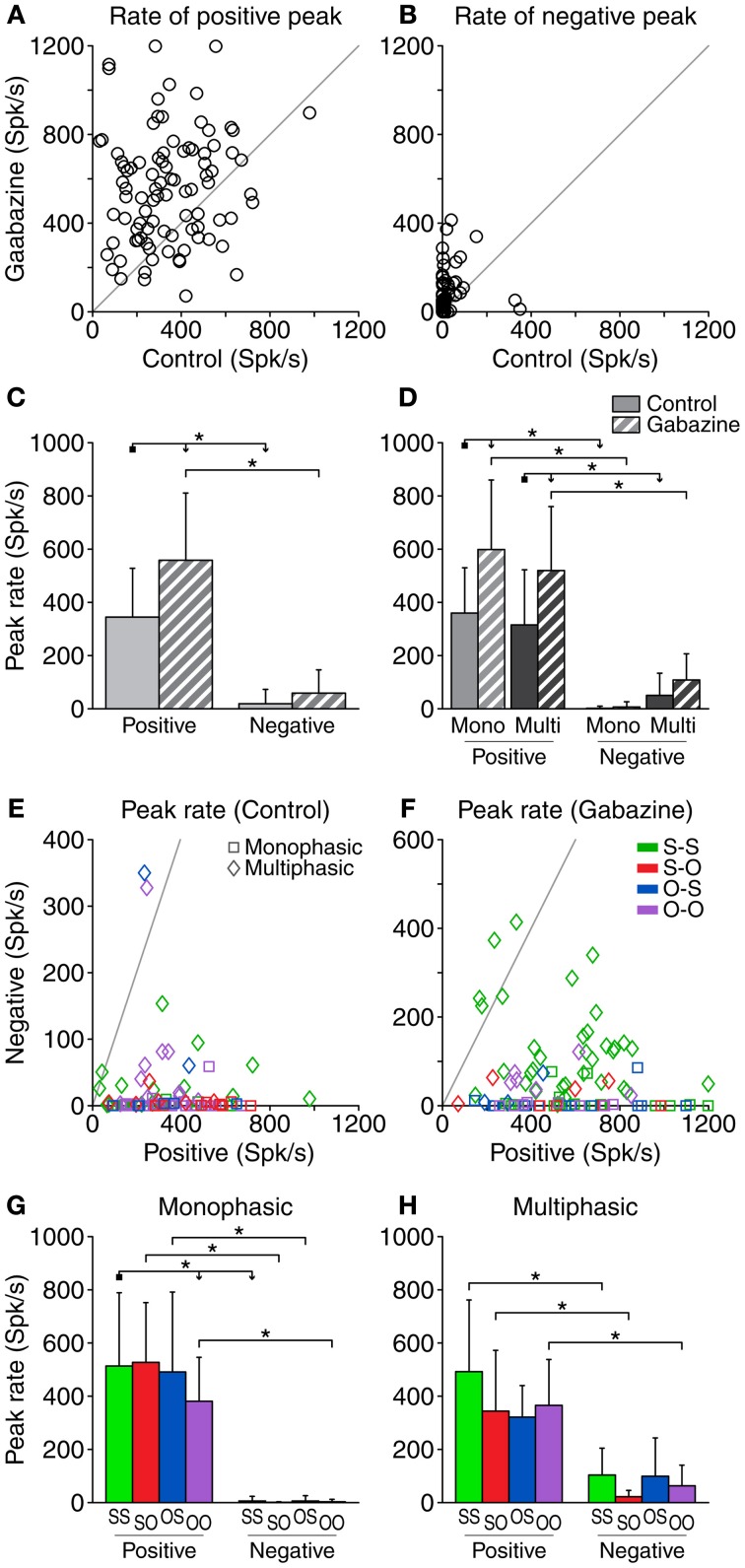
**Peak firing rate of the DS for each neuron.** The most positive (positive peak) and most negative (negative peak) values of the DS for each neuron are represented here in absolute value. The effect of removing GABA_A_ inhibition is depicted in **(A)** and **(B)**. Both the positive peak rates **(A)** and the negative **(B)** tend to increase due to the application of gabazine, to the point of the appearance of negative parts in the DS where there was previously none in the control condition **(B)**. **(C)** Mean and standard deviation of the peak firing rate for all the cases pooled together, in the control (solid fill) and gabazine (pattern fill) conditions. **(D)** Mean and standard deviation of the peak firing rate grouped by DS type. In the control condition **(E)**, the peak positive DS extended over a wide range of values, while the negative peak was limited to much smaller rates. During the gabazine condition **(F)**, both positive and negative peaks became slightly larger. In almost every case, the positive peak was much larger than the negative peak. The axes in these panels have been plotted at different scales for a better visualization of the data. **(G)** Mean and standard deviation of the different firing pattern groups for the monophasic or **(H)** multiphasic units, including units in both the control and gabazine conditions. *Mono*, monophasic; *Multi*, multiphasic. *S-S*, standard and deviant sustained; *S-O*, standard sustained and deviant onset; *O-S*, standard onset and deviant sustained; *O-O*, standard and deviant onset. Asterisks indicate *p* < 0.05.

Similarly to the area of the DS, we quantified the peak rate of firing for the positive and negative portions of the DS (Figures 5A,B). The average peak rate of the positive portions was 344.4 ± 183.9 spk/s in the control condition and 558.2 ± 252.7 spk/s in the gabazine condition (Figure 5A). Gabazine caused an increment of the peak firing rate of the positive DS portions in 77% of the cases (71/92) and a decrement in the resting 23% (21/92). Considering all the cases, the average increment of the peak firing rate of the positive DS parts during the gabazine condition was 213.8 ± 293.9 spk/s. The peak firing rate of the negative parts was 18.9 ± 54.2 spk/s in the control condition, and increased to 58.5 ± 87.7 spk/s during the gabazine condition (Figure 5B). Fifty-eight percent of the cases (53/92) showed an increment of the peak firing rate of the negative DS portions during the gabazine condition, while 20% (18/92) showed a decrement. In 18 cases (20%), there was no negative part during either the control or the gabazine conditions. Taking all the cases into account, the average increment of the peak firing rate of the negative parts during the gabazine condition was 39.6 ± 93.6 spk/s.

When considering all the cases together, the average peak firing rate of the positive parts was significantly larger than that of the negative parts, both in the control and gabazine conditions. In addition, gabazine had a significant effect on the rate of the positive portions of the DS, but not on the negative ones (Figure 5C). The analysis of the peak firing rate grouping the data by DS and firing pattern types (Figure 5D) was similar to the case of the area of the DS parts (Figure 4D), with the exceptions that now there is a significant difference between the positive and negative peak firing rates for multiphasic units in the control condition, and the positive peak firing rates of monophasic and multiphasic units in the gabazine condition are not significantly different.

The DS did not have a negative portion in 36 cases during the control condition and in 29 during the gabazine condition. In a large majority of the cases showing a negative part, the peak firing rate of the positive part was larger than that of the negative part, both in the control (95%, 53/56) and the gabazine (94%, 59/63) conditions (Figures 5E,F).

The peak firing rate of the positive DS parts was larger than that of the negative parts for all the firing pattern groups, both for the monophasic and the multiphasic neurons, with the only exception of the O-S group of the multiphasic units (Figures 5G,H). For each DS type (monophasic or multiphasic) and DS part (positive or negative), the mean peak firing rate was quite similar among all firing pattern groups, with the exception of the peak firing rate of the positive DS parts of the monophasic units, where there was a significant difference between the S-S and O-O groups.

### Timing of the difference signal

**Figure 6:**
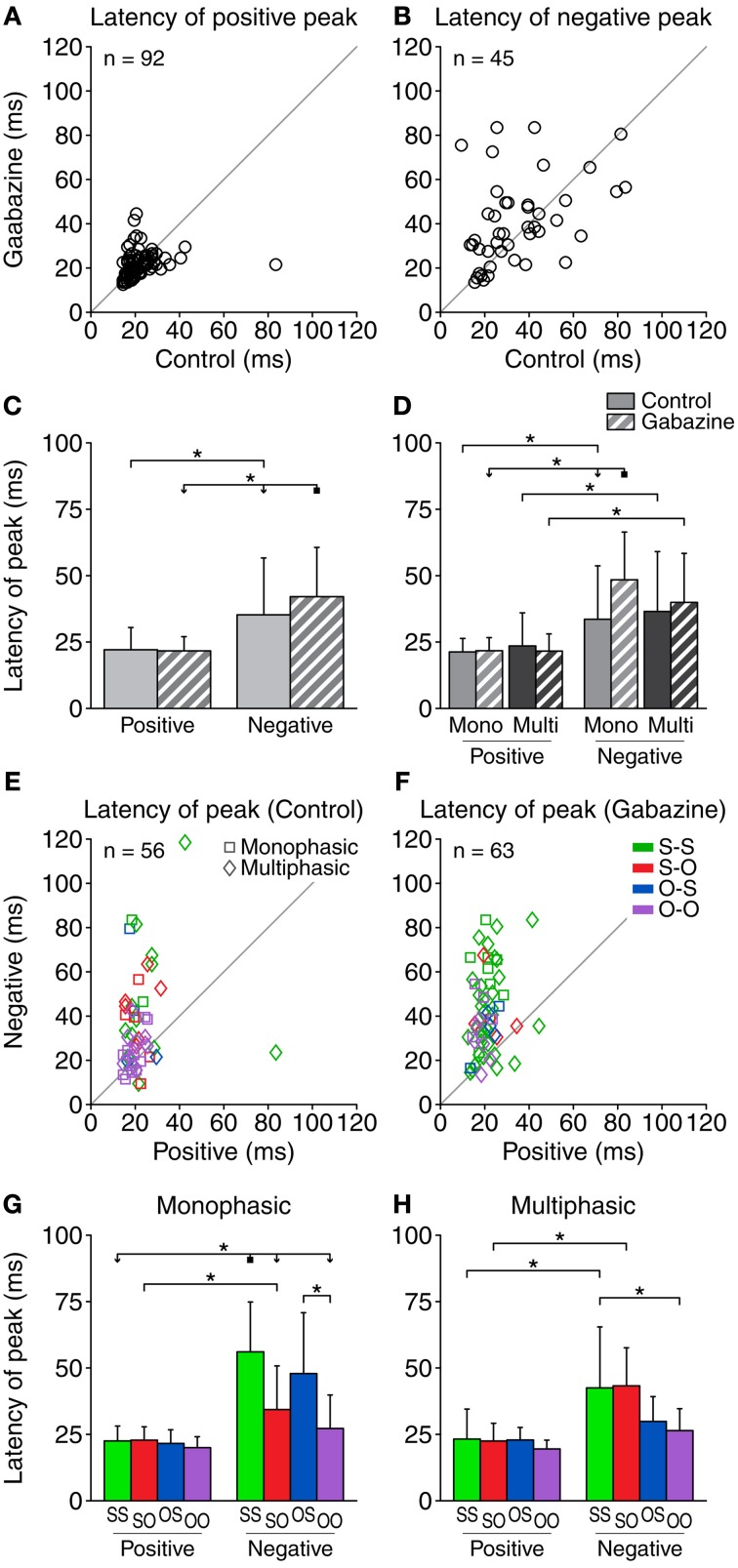
**The latency of the positive DS peak was relatively short (10–40 ms) and was little affected by gabazine (A), while the latency of the negative peak extended over a longer range of values (B).** Some of the cases did not contain a negative part of the DS, so the corresponding latencies could not be calculated. The number of cases plotted is indicated inside each panel. **(C)** Mean and standard deviation of the latency of the peaks for all the cases pooled together, in the control (solid fill) and gabazine (pattern fill) conditions. **(D)** Mean and standard deviation of the latency of the peaks, grouped by DS type. In both the control **(E)** and the gabazine **(F)** condition, the latency of the positive DS peak was shorter than the latency of the negative peak for most neurons. **(G)** Mean and standard deviation of the different firing pattern groups for the monophasic or **(H)** multiphasic units, including units in both the control and gabazine conditions. *Mono*, monophasic; *Multi*, multiphasic. *S-S*, standard and deviant sustained; *S-O*, standard sustained and deviant onset; *O-S*, standard onset and deviant sustained; *O-O*, standard and deviant onset. Asterisks indicate *p* < 0.05.

Regarding the timing of the different parts of the DS, we calculated the latency of the DS peaks (Figure 6). The average latency of the positive peaks was very similar in the control (22.1 ± 8.4 ms) and the gabazine (21.7 ± 5.7 ms) conditions (Figure 6A). The application of gabazine caused an increment of the latency of the positive peak on 39% of the cases (36/92), while 51% of the cases (47/92) showed a decrement. Taking all the cases into account, the average increment of the latency of the positive peak during the gabazine condition was −0.4 ± 9.1 ms. The average latency of the negative peaks (Figure 6B) was longer in the control condition (35.3 ± 21.4 ms), and even longer in the gabazine condition (42.1 ± 18.6 ms). Only 45 neurons showed a negative portion in both the control and gabazine conditions. Of those, 47% of the cases (21/45) showed an increment of the latency of the negative peak during the gabazine condition, and 44% of the cases (20/45) showed a decrement. Taking these 45 cases into account, the average increment of the latency of the negative peak during the gabazine condition was 6.1 ± 20.7 ms. Additionally, the individual latencies were more dispersed.

When considering all the cases together, the latency of the negative peaks was significantly longer than the latency of the positive peaks in both the control and gabazine conditions. Only for the negative peaks, the latency was significantly affected by gabazine (Figure 6C). When grouping the data by DS and firing pattern type (Figure 6D), we found that the latency of the negative peaks was significantly longer than the latency of the positive peaks for both the monophasic and the multiphasic groups, in both control and gabazine conditions. Qualitatively similar results have been obtained for the median latency (data not shown).

Gabazine increased the number of cases where the latency of the positive peak was shorter than the negative from 73% (41/56; in 36 cases there was no negative part) of the cases during the control condition, to 90% (57/63; in 29 cases there was no negative part) during the application of gabazine (Figures 6E,F).

The latency of the positive DS peaks did not differ among firing pattern groups (Figures 6G,H). The latency of the negative DS peaks was significantly larger for the S-S and the S-O groups, but not for the others.

### Probability of significant difference signal

**Figure 7:**
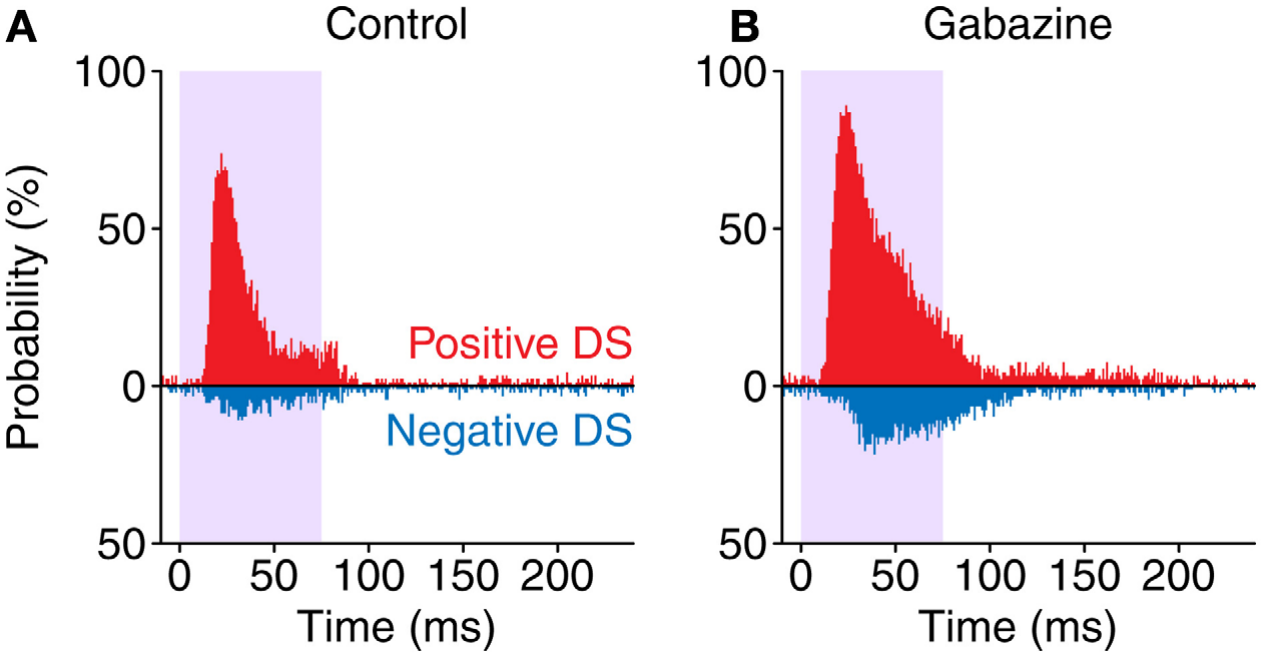
**Probability of occurrence of a positive (red) DS value or a negative one (blue) along time.** Note how in the gabazine condition **(B)** the probabilities are higher and skewed toward longer latencies, compared to the control condition **(A)**.

Since we found that most neurons presented both positive and negative portions on their DS, we calculated the probability of observing a positive or negative DS value for each bin of the PSTH (Figure 7). In the control condition, the maximum probability of evoking a positive DS was 74%, with a latency of 21.5 ms after the stimulus onset. On the other hand, the maximum probability of a negative DS was only 11% and happened later, with a latency of 30.5 ms. The application of gabazine caused an increment of these figures, with a maximum positive DS probability of 89%, 23.5 ms after the onset of the stimulus, and maximum negative DS probability of 22% with a latency of 38.5 ms. When considering a window of 10–115 ms after the stimulus onset (which encompasses approximately the evoked portion of the responses), during the control condition the occurrence of a positive DS was 4.09 times more likely than a negative one. On the other hand, during the gabazine condition this ratio was reduced to 2.96. This way, inhibition increases the contrast between the positive and the negative portions of the DS in neurons showing strong SSA. These probability functions remark that SSA in the IC is stronger at the beginning of the stimulus, as a consequence of the shorter latencies in response to deviants. Nevertheless, even highly adapting neurons (such as those in this sample) may still momentarily respond better to standards than to deviants afterwards.

## Discussion

In this study we report that even in a sample comprised of neurons showing very strong SSA (i.e., they respond more intensely to deviant stimuli overall), a subset of the neurons (one third of the cases in the control condition) contain portions that responded significantly better to the standard stimuli than to the deviants. Furthermore, the blockade of GABA_A_ receptors increases the number of neurons showing portions that responded better to the standard stimuli, indicating that in the IC, inhibition refines the responses to SSA by sharpening or “cleaning” the “noise” produced by the portions that respond better to standards.

Due to the large variability in the timing of the negative portions of the DS (Figure 6), when considering the population as a whole these parts get averaged out (Figures 2A,B) and become unnoticed, both before and during application of gabazine. On the other hand, the smaller variability of the latencies of the positive portions contributes to the large peak observed in the averaged representations, like in Figures 2A,B. Other previous studies describing the average response in form of PSTH in the IC (Malmierca et al., 2009a; Zhao et al., 2011; Duque et al., 2012), auditory thalamus (Yu et al., 2009; Antunes et al., 2010; Bäuerle et al., 2011), and cortex (Ulanovsky et al., 2003; von der Behrens et al., 2009; Farley et al., 2010) have shown similar results at the population level, but they have not studied the influence of inhibition. It is only when analyzing the responses of individual neurons when these negative portions of the DS become noticeable. As a result, other types of measurements that depend of the global activity of a large number of neurons (like evoked potential recordings) are unlikely to reflect these particular details from the individual neurons. However, the negative DS at the single neuron level contributes to reduce the magnitude of the population signal at long latencies, narrowing the onset response, which can improve temporal accuracy.

The presence of negative portion in the DS was closely related to the type of firing pattern of the neuron in response to standard and deviant stimuli (Figure 2D). Taking together the control and gabazine conditions, the group with the largest proportion of multiphasic units was S-S (58%, 48/83), followed by S-O (44%, 10/23), O-O (29%, 16/55) and O-S (26%, 6/23). This indicates that a sustained response to deviants facilitates the occurrence of negative DS parts. The larger number of multiphasic units found during the application of gabazine appears to be related to the lengthening of the neuronal responses caused by the reduction of the incoming inhibition, which increased the number of units classified as S-S. This type of firing pattern stands out from the others, as the neurons in this group are among the ones with the largest positive and negative DS duration, area, and peak firing rate, as well as the largest negative DS latency.

The latency of the negative DS part was generally longer than the latency of the positive DS part, both when measuring the latency of the peak DS (Figure 6) or the latency of the median spike (not shown). This is especially notable for the neurons that respond with a sustained firing rate to both standards and deviants (group S-S), since the longer duration of the responses allows for a better temporal separation of the positive and negative parts of the DS. However, even in the least favorable case, which the shortest responses to both standards and deviants (group O-O), the average latency of the negative parts of the DS is longer than the latency of the positive parts (Figures 6G,H), although the differences are not significant. The latencies of the positive parts (~22 ms on average for the peak) fall within the range of onset responses in the cortical areas of the IC (e.g., Lumani and Zhang, 2010). The longer latencies of the negative DS parts, observed in the majority of neurons, reflect the differential responses to deviant and standard stimuli. A faster response to deviants is responsible for the early, positive DS peak, while a slower response to the standards produces the negative DS peak. These differences in the latency of the response to the stimuli with different probabilities have been reported previously in the IC (Malmierca et al., 2009a; Lumani and Zhang, 2010; Zhao et al., 2011; Duque et al., 2012; Pérez-González et al., 2012).

### Comparison to other studies

Several previous studies have reported the time course of neuronal responses related to SSA. Due to the lack of testing of the contribution of inhibition to SSA in previous studies, the following comparisons will refer only to our results in the control condition.

In an initial report of SSA in the IC, we showed (Malmierca et al., 2009a) that the PSTH-based population DS was mostly positive in different stimulation conditions, with peak latencies of 25–30 ms. We also found that the responses to deviant stimuli had shorter latencies than the responses to standard stimuli, which agrees with the present study. This finding was also reported in the dorsal cortex of the IC in rats (Lumani and Zhang, 2010). A study of the dynamics of adaptation in neurons through the IC showed that the general shape of the population DS was maintained at interstimulus intervals of up to 2 s, which is rather slow for this nucleus (Ayala and Malmierca, 2012). In the dorsal cortex of the IC, SSA was also reflected in the negative deflection of LFPs (Patel et al., 2012). The time course of the difference between the LFPs evoked by deviants and standards was comparable to that of the PSTH-based population DS, since in both cases the magnitude of the responses to deviants was consistently larger than that of the standards. In other words, neither the LFP nor the population DS did reflect the negative DS portions that we have shown here in single neurons, suggesting that they are averaged out in population measures.

In the medial geniculate body of the thalamus (Antunes et al., 2010), the population DS seems to be quite similar to that reported here for the IC. Some of the figures in Bäuerle et al. (2011) show the DS for some individual units, and several cases show consistent negative DS portions, although they do not discuss it. In any case, their sample of neurons from the ventral nucleus of the medial geniculate body showed a small degree of SSA, so that kind of response is as expected.

In the auditory cortex of an anesthetized preparation (Ulanovsky et al., 2003; Farley et al., 2010; Taaseh et al., 2011; Yaron et al., 2012), the first place where auditory SSA was described, the population DS seems to be more sustained. This contrasts with awaken animal preparations, were the neuronal responses are onset-like (von der Behrens et al., 2009). Due to this firing pattern, the calculated population DS in the cortex of an awaken preparation resulted to be significant at latencies of 12–28 ms, which is comparable to the latency of the positive peaks found in the IC (22.1 ± 8.4 ms).

### Effect of gabazine

Blocking GABA_A_ receptors in the IC has been reported to increase the firing rates in response to stimuli, modify the response patterns and reduce the first spike latency in response to deviant stimuli (Pérez-González et al., 2012). By making the responses to standard relatively slower and more sustained than the responses to deviants, these effects may explain the stronger negative DS that is found during the gabazine condition, as well as the larger percentage of the overall response that is negative during the gabazine condition. Furthermore, this may be the reason of the increment in the number of multiphasic neurons during this condition.

As GABA is the main inhibitory neurotransmitter in the dorsal and lateral cortices of the IC, as indicated by the much larger numbers of GABAergic than glycinergic puncta (Merchán et al., 2005), the application of gabazine reflects a situation where inhibition is largely reduced and excitation is the most prominent driving force of these neurons. Based on these findings, we propose that excitation alone generates responses to deviant and standard stimuli that are quite different in shape and latency, resulting in DS that are more prone to variability during the response (e.g., changing from positive to negative). In addition to providing a mechanism of gain control that modulates the strength of SSA (Pérez-González et al., 2012), inhibition would reduce this variability of the responses, affecting mostly the negative parts of the DS, and thus sharpening the response to SSA.

In conclusion, the variability of the time course of the responses of neurons that show SSA, responsible for negative DS portions even in neurons that overall prefer deviant stimuli, is reduced by GABA and seems to be only evident on a single neuron level. Most studies on SSA tend to report aggregated data at the population level, which obscures this variability. Our study emphasizes the importance of reporting the details of the responses of single neurons, since paying attention only to the big picture may result in the omission of important functional features.

#### Conflict of interest statement

The authors declare that the research was conducted in the absence of any commercial or financial relationships that could be construed as a potential conflict of interest.

## References

[B1] Anderson L. A. Christianson G. B. Linden J. F. (2009). Stimulus-specific adaptation occurs in the auditory thalamus. J. Neurosci. 29, 7359–7363 10.1523/JNEUROSCI.0793-09.2009 19494157 PMC6666468

[B1a] Anderson L. A. Malmierca M. S. (2012). The effect of auditory cortex deactivation on stimulus-specific adaptation in the inferior colliculus of the rat. Eur. J. Neurosci. [Epub ahead of print]. 10.1111/ejn.12018 23121128

[B2] Antunes F. M. Malmierca M. S. (2011). Effect of auditory cortex deactivation on stimulus-specific adaptation in the medial geniculate body. J. Neurosci. 31, 17306–17316 10.1523/JNEUROSCI.1915-11.2011 22114297 PMC6623836

[B3] Antunes F. M. Nelken I. Covey E. Malmierca M. S. (2010). Stimulus-specific adaptation in the auditory thalamus of the anesthetized rat. PLoS ONE 5:e14071 10.1371/journal.pone.0014071 21124913 PMC2988819

[B3a] Ayala Y. A. Malmierca M. S. (2012). Stimulus-specific adaptation and deviance detection in the inferior colliculus. Front. Neural Circuits 6:89 10.3389/fncir.2012.00089 PMC3547232 23335883

[B4] Bäuerle P. von der Behrens W. Kössl M. Gaese B. H. (2011). Stimulus-specific adaptation in the gerbil primary auditory thalamus is the result of a fast frequency-specific habituation and is regulated by the corticofugal system. J. Neurosci. 31, 9708–9722 10.1523/JNEUROSCI.5814-10.2011 21715636 PMC6623171

[B5] Caspary D. M. Palombi P. S. Hughes L. F. (2002). GABAergic inputs shape responses to amplitude modulated stimuli in the inferior colliculus. Hear. Res. 168, 163–173 10.1016/S0378-5955(02)00363-5 12117518

[B6] Casseday J. H. Ehrlich D. Covey E. (1994). Neural tuning for sound duration: role of inhibitory mechanisms in the inferior colliculus. Science 264, 847–850 10.1126/science.8171341 8171341

[B7] Casseday J. H. Ehrlich D. Covey E. (2000). Neural measurement of sound duration: control by excitatory-inhibitory interactions in the inferior colliculus. J. Neurophysiol. 84, 1475–1487 10980020 10.1152/jn.2000.84.3.1475

[B8] Covey E. Kauer J. A. Casseday J. H. (1996). Whole-cell patch-clamp recording reveals subthreshold sound-evoked postsynaptic currents in the inferior colliculus of awake bats. J. Neurosci. 16, 3009–3018 8622130 10.1523/JNEUROSCI.16-09-03009.1996 PMC6579070

[B8a] Duque D. Pérez-González D. Ayala Y. A. Palmer A. R. Malmierca M. S. (2012). Topographic distribution, frequency, and intensity dependence of stimulus-specific adaptation in the inferior colliculus of the rat. J. Neurosci. 32, 17762–17774 10.1523/JNEUROSCI.3190-12.2012 23223296 PMC6621662

[B9] Escera C. Alho K. Winkler I. Näätänen R. (1998). Neural mechanisms of involuntary attention to acoustic novelty and change. J. Cogn. Neurosci. 10, 590–604 9802992 10.1162/089892998562997

[B10] Farley B. J. Quirk M. C. Doherty J. J. Christian E. P. (2010). Stimulus-specific adaptation in auditory cortex is an NMDA-independent process distinct from the sensory novelty encoded by the mismatch negativity. J. Neurosci. 30, 16475–16484 10.1523/JNEUROSCI.2793-10.2010 21147987 PMC6634869

[B11] Fritz J. B. Elhilali M. David S. V. Shamma S. A. (2007). Auditory attention–focusing the searchlight on sound. Curr. Opin. Neurobiol. 17, 437–455 10.1016/j.conb.2007.07.011 17714933

[B11a] Gittelman J. X. Wang L. Colburn H. S. Pollak G. D. (2012). Inhibition shapes response selectivity in the inferior colliculus by gain modulation. Front. Neural Circuits 6:67 10.3389/fncir.2012.00067 23024629 PMC3444759

[B12] Grimm S. Escera C. Slabu L. Costa-Faidella J. (2011). Electrophysiological evidence for the hierarchical organization of auditory change detection in the human brain. Psychophysiology 48, 377–384 10.1111/j.1469-8986.2010.01073.x 20636288

[B12a] Gutfreund Y. (2012). Stimulus-specific adaptation, habituation and change detection in the gaze control system. Biol. Cybern. 106, 657–668 10.1007/s00422-012-0497-3 22711216

[B13] Ingham N. J. McAlpine D. (2005). GABAergic inhibition controls neural gain in inferior colliculus neurons sensitive to interaural time differences. J. Neurosci. 25, 6187–6198 10.1523/JNEUROSCI.0146-05.2005 15987948 PMC6725068

[B14] Isaacson J. S. Scanziani M. (2011). How inhibition shapes cortical activity. Neuron 72, 231–243 10.1016/j.neuron.2011.09.027 22017986 PMC3236361

[B15] Jääskeläinen I. P. Ahveninen J. Bonmassar G. Dale A. M. Ilmoniemi R. J. Levänen S. (2004). Human posterior auditory cortex gates novel sounds to consciousness. Proc. Natl. Acad. Sci. U.S.A. 101, 6809–6814 10.1073/pnas.0303760101 15096618 PMC404127

[B16] Koch U. Grothe B. (1998). GABAergic and glycinergic inhibition sharpens tuning for frequency modulations in the inferior colliculus of the big brown bat. J. Neurophysiol. 80, 71–82 9658029 10.1152/jn.1998.80.1.71

[B17] Kurt S. Crook J. M. Ohl F. W. Scheich H. Schulze H. (2006). Differential effects of iontophoretic *in vivo* application of the GABA_A_-antagonists bicuculline and gabazine in sensory cortex. Hear. Res. 212, 224–235 10.1016/j.heares.2005.12.002 16442250

[B18] LeBeau F. E. Malmierca M. S. Rees A. (2001). Iontophoresis *in vivo* demonstrates a key role for GABA_A_ and glycinergic inhibition in shaping frequency response areas in the inferior colliculus of guinea pig. J. Neurosci. 21, 7303–7312 11549740 10.1523/JNEUROSCI.21-18-07303.2001 PMC6762982

[B19] LeBeau F. E. Rees A. Malmierca M. S. (1996). Contribution of GABA- and glycine-mediated inhibition to the monaural temporal response properties of neurons in the inferior colliculus. J. Neurophysiol. 75, 902–919 8714663 10.1152/jn.1996.75.2.902

[B20] Lumani A. Zhang H. (2010). Responses of neurons in the rat's dorsal cortex of the inferior colliculus to monaural tone bursts. Brain Res. 1351, 115–129 10.1016/j.brainres.2010.06.066 20615398

[B21] Malmierca M. S. (2003). The structure and physiology of the rat auditory system: an overview. Int. Rev. Neurobiol. 56, 147–211 14696313 10.1016/s0074-7742(03)56005-6

[B22] Malmierca M. S. Blackstad T. W. Osen K. K. (2011). Computer-assisted 3-D reconstructions of Golgi-impregnated neurons in the cortical regions of the inferior colliculus of rat. Hear. Res. 274, 13–26 10.1016/j.heares.2010.06.011 20600744

[B23] Malmierca M. S. Blackstad T. W. Osen K. K. Karagulle T. Molowny R. L. (1993). The central nucleus of the inferior colliculus in rat: a Golgi and computer reconstruction study of neuronal and laminar structure. J. Comp. Neurol. 333, 1–27 10.1002/cne.903330102 7688006

[B24] Malmierca M. S. Cristaudo S. Pérez-González D. Covey E. (2009a). Stimulus-specific adaptation in the inferior colliculus of the anesthetized rat. J. Neurosci. 29, 5483–5493 10.1523/JNEUROSCI.4153-08.2009 19403816 PMC2715893

[B25] Malmierca M. S. Hernandez O. Antunes F. M. Rees A. (2009b). Divergent and point-to-point connections in the commissural pathway between the inferior colliculi. J. Comp. Neurol. 514, 226–239 10.1002/cne.21997 19296464 PMC2771101

[B25a] Malmierca M. S. Izquierdo M. A. Cristaudo S. Hernández O. Pérez-González D. Covey E. (2008). A discontinuous tonotopic organization in the inferior colliculus of the rat. J. Neurosci. 28, 4767–4776 10.1523/JNEUROSCI.0238-08.2008 18448653 PMC2440588

[B25b] Malmierca M. S. Ryugo D. K. (2011). Descending connections of auditory cortex to the midbrain and brainstem, in The Auditory Cortex, eds Winer and J. A. Schreiner C. E. (New York, NY: Springer-Verlag), 189–208

[B25c] Malmierca M. S. Ryugo D. K. (2012). Sensory systems: Auditory, in The Mouse Nervous System, eds Watson C. Paxinos G. Puelles L. (San Diego, CA: Academic Press), 607–645

[B26] Merchán M. Aguilar L. A. Lopez-Poveda E. A. Malmierca M. S. (2005). The inferior colliculus of the rat: quantitative immunocytochemical study of GABA and glycine. Neuroscience 136, 907–925 10.1016/j.neuroscience.2004.12.030 16344160

[B27] Näätänen R. (1992). Attention and Brain Function. Hillsdale, NJ: Erlbaum

[B28] Nelken I. Ulanovsky N. (2007). Mismatch negativity and stimulus-specific adaptation in animal models. J. Psychophysiol. 21, 214–223

[B29] Oliver D. L. Shneiderman A. (1991). The anatomy of the inferior colliculus. A cellular basis for integration of monaural and binaural information, in Neurobiology of Hearing, eds Altschuler R. A. Bobbin R. P. Clopton B. M. Hoffmann D. W. (New York, NY: Raven Press), 195–222

[B30] Palombi P. S. Caspary D. M. (1996). GABA inputs control discharge rate primarily within frequency receptive fields of inferior colliculus neurons. J. Neurophysiol. 75, 2211–2219 8793735 10.1152/jn.1996.75.6.2211

[B31] Patel C. R. Redhead C. Cervi A. L. Zhang H. (2012). Neural sensitivity to novel sounds in the rat's dorsal cortex of the inferior colliculus as revealed by evoked local field potentials. Hear. Res. 286, 41–54 10.1016/j.heares.2012.02.007 22406035

[B32] Pérez-González D. Hernández O. Covey E. Malmierca M. S. (2012). GABA_A_-mediated inhibition modulates stimulus-specific adaptation in the inferior colliculus. PLoS ONE 7:e34297 10.1371/journal.pone.0034297 22479591 PMC3315508

[B33] Pérez-González D. Malmierca M. S. Covey E. (2005). Novelty detector neurons in the mammalian auditory midbrain. Eur. J. Neurosci. 22, 2879–2885 10.1111/j.1460-9568.2005.04472.x 16324123

[B34] Rees A. (1990). A closed field sound system for auditory neurophysiology. J. Physiol. 430, 6

[B35] Rees A. Sarbaz A. Malmierca M. S. LeBeau F. E. (1997). Regularity of firing of neurons in the inferior colliculus. J. Neurophysiol. 77, 2945–2965 9212248 10.1152/jn.1997.77.6.2945

[B36] Rose D. Blakemore C. (1974). Effects of bicuculline on functions of inhibition in visual cortex. Nature 249, 375–377 4842746 10.1038/249375a0

[B37] Sivaramakrishnan S. Sterbing-D'Angelo S. J. Filipovic B. D'Angelo W. R. Oliver D. L. Kuwada S. (2004). GABA(A) synapses shape neuronal responses to sound intensity in the inferior colliculus. J. Neurosci. 24, 5031–5043 10.1523/JNEUROSCI.0357-04.2004 15163696 PMC6729375

[B38] Slabu L. Escera C. Grimm S. Costa-Faidella J. (2010). Early change detection in humans as revealed by auditory brainstem and middle-latency evoked potentials. Eur. J. Neurosci. 32, 859–865 10.1111/j.1460-9568.2010.07324.x 20626459

[B38a] Taaseh N. Yaron A. Nelken I. (2011). Stimulus-specific adaptation and deviance detection in the rat auditory cortex. PLoS ONE 6:e23369 10.1371/journal.pone.0023369 21853120 PMC3154435

[B39] Ulanovsky N. Las L. Farkas D. Nelken I. (2004). Multiple time scales of adaptation in auditory cortex neurons. J. Neurosci. 24, 10440–10453 10.1523/JNEUROSCI.1905-04.2004 15548659 PMC6730303

[B40] Ulanovsky N. Las L. Nelken I. (2003). Processing of low-probability sounds by cortical neurons. Nat. Neurosci. 6, 391–398 10.1038/nn1032 12652303

[B41] von der Behrens W. Bäuerle P. Kössl M. Gaese B. H. (2009). Correlating stimulus-specific adaptation of cortical neurons and local field potentials in the awake rat. J. Neurosci. 29, 13837–13849 10.1523/JNEUROSCI.3475-09.2009 19889995 PMC6666711

[B42] Winkler I. Denham S. L. Nelken I. (2009). Modeling the auditory scene: predictive regularity representations and perceptual objects. Trends Cogn. Sci. 13, 532–540 10.1016/j.tics.2009.09.003 19828357

[B43] Wu S. H. Ma C. L. Kelly J. B. (2004). Contribution of AMPA, NMDA, and GABA(A) receptors to temporal pattern of postsynaptic responses in the inferior colliculus of the rat. J. Neurosci. 24, 4625–4634 10.1523/JNEUROSCI.0318-04.2004 15140934 PMC6729405

[B43a] Yaron A. Hershenhoren I. Nelken I. (2012). Sensitivity to complex statistical regularities in rat auditory cortex. Neuron 76, 603–615 10.1016/j.neuron.2012.08.025 23141071

[B44] Yu X. J. Xu X. X. He S. He J. (2009). Change detection by thalamic reticular neurons. Nat. Neurosci. 12, 1165–1170 10.1038/nn.2373 19684591

[B45] Zhang H. Kelly J. B. (2003). Glutamatergic and GABAergic regulation of neural responses in inferior colliculus to amplitude-modulated sounds. J. Neurophysiol. 90, 477–490 10.1152/jn.01084.2002 12660357

[B46] Zhao L. Liu Y. Shen L. Feng L. Hong B. (2011). Stimulus-specific adaptation and its dynamics in the inferior colliculus of rat. Neuroscience 181, 163–174 10.1016/j.neuroscience.2011.01.060 21284952

